# Comparison of the Calibration Standards of Three Commercially Available Multiplex Kits for Human Cytokine Measurement to WHO Standards Reveals Striking Differences

**DOI:** 10.4137/bmi.s660

**Published:** 2008-04-18

**Authors:** Andreas Nechansky, Susanne Grunt, Ivan M. Roitt, Ralf Kircheis

**Affiliations:** 1 Vela Laboratories GmbH, Brunner Strasse 69/Obj. 3, 1230 Wien, Austria; 2 AVIR Green Hills Biotechnology AG, Gersthofer Strasse 29–31, 1180 Wien, Austria; 3 University College London, Dep. Immunology, London, United Kingdom

**Keywords:** cytokines, biomarker, multiplex, WHO standards

## Abstract

Serum parameters as indicators for the efficacy of therapeutic drugs are currently in the focus of intensive research. The induction of certain cytokines (or cytokine patterns) is known to be related to the status of the immune response e.g. in regulating the T_H_1/T_H_2 balance. Regarding their potential value as surrogate parameters in clinical trials and subsequently for the assignment of treatment efficacy, the accurate and reliable determination of cytokines in patient serum is mandatory. Because serum samples are precious and limited, test methods—like the xMAP multiplex technology—that allow for the simultaneous determination of a variety of cytokines from only a small sample aliquot, can offer great advantages.

We here have compared multiplex kits from three different manufactures and found striking differences upon standardizing using WHO standards for selected cytokines. We therefore extended our xMAP multiplex measurements investigations to an *ex-vivo* situation by testing serum samples and found that the cytokine amounts measured was critically influenced by the actual kit used. The presented data indicate that statements regarding the quantitive determination of cytokines—and therefore their use as biomarkers—in serum samples have to be interpreted with caution.

## Introduction

For the investigation of cytokines involved in immune responses, multiplex kits have been shown to be of particular value (Jiang et al. 2003; Lagrelius et al. 2006; Panelli et al. 2004; Kircheis et al. 2006). One reason for this is that cytokine patterns rather than single cytokines have a pivotal importance in defining the balance between e.g. activation versus tolerization, or T_H_1 versus T_H_2 responses (Prabhakar et al. 2004; Kircheis et al. 1998) or in the angiogenesis process (Piperi et al. 2005). Although multiplex kits are usually more expensive than in-house designed assays, they are convenient because they allow performing a multi-parameter analysis within a short time period using only minute amounts of precious serum samples (Hildesheim et al. 2003; DeJager et al. 2003; Giavedoni, 2005). In many research publications the use of such kits is only briefly described in the material and methods section using statements such as: “… determined plasma concentration of cytokines, including IL1β, IL6 and TNFα, using a mouse Luminex kit …” (Cai et al. 2005). In contrast in-house designed assays usually require a detailed description and therefore the quality can be—at least in part—judged based on the supplied method description.

The vaccine-mediated induction of certain cytokines by active anti-cancer immuntherapy may be a valuable predictor for the subsequent treatment efficacy (for review see Dranoff, 2004). For example, TNFα is selectively cytotoxic to a variety of transformed cells and many actions of TNFα occur in combination with other cytokines as part of the cytokine network (Ruddle, 1992). IFNγ is involved in nearly all phases of immune responses including activation, growth and differentiation of T-cells, B-cells, macrophages, and NK cells. Additionally, IFNγ enhances MHC expression on antigen-presenting cells (Schiller et al. 1990). IL-2 and IL-4 are representative cytokines involved in T-cell activation, and the balance between Th1 and Th2 is largely determined by the balance between IFNγ and IL-4, respectively. Furthermore, the cytokines IL-2, IL-4, TNFα and IFNγ have been shown to inhibit tumor-induced angiogenesis in some animal models by inhibition of tumor stroma formation (Prabhakar et al. 2004; Blankenstein, 2005). In order for such cytokines to be used as surrogate clinical parameters, their accurate determination is mandatory because usually threshold concentrations have to be reached for effective cytokine actions (Kircheis et al. 1992; Schmidt et al. 1995; Yang et al. 2004).

To minimize sample consumption during cytokine determination, xMAP multiplex technology was applied which uses spectrally addressable polystyrene beads as array elements to enable the simultaneous analysis of up to one-hundred analytes in only a few microliters of sample. By coupling the cytokine specific capture antibody to a specific bead in combination with a phycoerythrin labeled cytokine specific detection antibody, the amounts of cytokines present in samples can be determined.

To get insight into the accuracy of commercially available test systems for the detection of INFγ, TNFα, IL-2 and IL-4, we have compared kits from different manufacturers, i.e. Biosource, Linco and Upstate by testing them according to the protocol provided by each manufacturer. INFγ, TNFα, IL-2 and IL-4 were chosen because they represent a well-documented panel of cytokines involved in regulating the immune system (Ruddle, 1992; Schiller et al. 1990; Blankenstein, 2005). Our experiments included the comparison of the three kit standards by measuring them with each kit (i.e. Biosource, Linco and Upstate, respectively) and the testing of the correlation of the kit standard with the WHO standard.

As an example, we tested the xMAP multiplex approach for measurement of cytokine release of Rhesus monkeys before and after immunization with the multi-component cancer vaccine Vela402 (previously IGN402). This vaccine has been shown to induce a significant IgG response against carrier protein and the attached carbohydrate epitopes (Kircheis et al. 2006; Kircheis et al. 2007).

Generally, our data demonstrate that the determination of the amount of various cytokines in serum samples using Luminex based multiplex technology is critically influenced by the actual kit used. In context of the increasing use of multiplexed kits in biomedical research and their potential application for surrogate markers in clinical trials, the results of the present study indicate that such data have to be interpreted with caution. Therefore, although the multiplex technology offers important advantages like the use of limited sample volume, the possibility of measuring a broader spectrum of biomarkers, reduced time and consumables costs and scalability (Ray et al. 2005), assay validation is of utmost importance for the acquisition of reliable data.

## Material and Methods

### WHO standards

Freeze dried recombinant human interferon gamma (IFNγ) reference (Cat. Nr. #Gxg01-902-535) was kindly provided by the National Institute of Allergy and Infectious Diseases (NIAID) reference reagent deposit operated by KamTek, MD, U.S.A. Freeze dried human natural tumour necrosis alpha (TNFα) reference (code 88/786), freeze dried human recombinant interleukin-4 (IL4) reference (code 88/656) and freeze dried human recombinant interleukin-2 (IL2) reference (code 86/504) were kindly provided by the National Institute for Biological Standards and Control (NIBSC), Hertfordshire, U.K. Each standard was reconstituted according to the accompanying NIAID or NIBSC datasheet, aliquoted and stored at −80 °C. One aliquot was used for each assay—left over material from the experiments was discarded.

### Quantitation of cytokine levels using xMAP technology

For cytokine determination, multiplex kits from Upstate (NY, U.S.A.), Biosource (Invitrogen, CA, U.S.A.) and Linco (MI, U.S.A.) were purchased and used according to the manufactures protocol. Briefly, the beads provided within each kit were incubated with buffer, kit standards or serum samples in a 96-well plate at room temperature. All incubations were performed at room temperature in the dark (covered with aluminum foil) on a plate shaker. Millipore multiscreen plates (Cat. Nr. #MABVN12) were used together with the Millipore filtration system (Cat. Nr. #MAVM0960R)— the vacuum suction during each washing step was set to ~200μl/5 seconds. For measurement, a Luminex IS 100 equipment was used. One-hundred events per bead region were counted (sample volume 50 μl) with the Double Discriminator gate set to 8000–15000. Data were evaluated applying a 5-parameter logistic curve fit using the StarStation software version 1.1. All specimens were tested in duplicates and the results are reported as the mean. The standards curve was generated by a 5-parameter logistic fit.

### Protocol: Upstate kit

According to ‘Multiple Cytokine detection protocol B’ the lyophilized standards (from 100 μl PBS, pH 7.4 with 10% BSA, 5% Trehalose, and 0.05% NaN_3_) were resuspended in 1 ml Serum Standard Diluent (proprietary formulation of animal protein and sera buffered with phosphate containing 0.1% NaN_3_). Briefly, to the pre-wetted wells, 50 μl of sample or standard was added followed by 25 μl sonicated bead solution. After incubating 2 hours, the wells were washed once with 50 μl Assay Buffer (PBS, 0.05% NaN_3_, 0.05% Tween-20, 1% BSA, pH 7.4) and the beads were resuspended in 75 μl Assay Buffer. Then, 25 μl Beadlyte biotinylated anti-human multicytokine antibodies were added to each well and incubated for 90 minutes. Then, 25 μl of diluted Streptavidin-Phycoerithrin were added to each well, vortexed and incubated for 30 minutes. Afterwards 25 μl Stop Solution were added, vortexed and incubated for 5 minutes. The liquid was removed by vacuum suction and the samples were resuspended in 125 μl Assay Buffer. The plate was gently vortexed, incubated on a shaker for 60 seconds and read on a Luminex IS 100 instrument.

### Protocol: Linco kit

All reagents were brought to room temperature. Standards were reconstituted with deionized water and further diluted with Assay Buffer (50 mM PBS, 25 mM EDTA, 0.08% NaN_3_, 0.05% Tween-20, 1% BSA, pH 7.4). To the pre-wetted wells, 25 μl of Assay Buffer were added, followed by 25 μl sample (to the wells representing background, standards and control, 25 μl Serum Matrix is added) and 25 μl beads. After incubation for 1 hour, the fluid was removed by vacuum suction. The plate was washed twice with Wash Buffer and remaining liquid from the bottom of the plate was removed by blotting the plate on paper towels. Then, 25 μl of Detection Antibody Cocktail was added and incubated for 30 minutes. After washing twice as described in the previous step, 100 μl Sheat Fluid was added to all wells and beads were resuspended by shaking for 5 minutes. Afterwards the plate was measured.

### Protocol: Biosource kit

To the pre-wetted wells, 25 μl antibody coated beads were added followed by 50 μl Incubation Buffer (proprietary formulation), 50 μl Dilution Buffer (proprietary formulation) and 50 μl serum sample (or spiked NHS). The standards were reconstituted and diluted in Assay Diluent (proprietary formulation). The plate was shaken for 2 hours and after washing twice, 100 μl biotinylated detection antibody were added and incubated for 1 hours. After two further washing steps, 100 μl of Streptavidin-Phycoerythrin were added and incubated for 30 minutes. The plate was washed three times and 100 μl Wash Buffer were added. The plate was then shaken for 30 seconds and measured.

### Comparison of the kit standard to the WHO reference

Human serum containing cytokines was mimicked by spiking the WHO standards into normal human serum (NHS). To get insight into the recovery rate of the spiked WHO standard using the standard curve obtained with the standards provided with each individual kit, the following experimental set-up was applied:

- for the Upstate kit, the Upstate cytokine standard was dissolved and diluted in Upstate Serum Standard Diluent (SSD) provided with the kit. The WHO standards were spiked into 1 ml of NHS (= patient matrix mimic) and diluted further with SSD. The assay was performed according to the Upstate protocol as described above.
- for the Linco Kit, the Linco cytokine standard were reconstituted with deionized water and further diluted with Assay Buffer. The WHO standards were spiked into 1 ml of NHS and diluted further with Assay Buffer. The assay was performed according to the Upstate protocol as described in above.
- for the Biosource Kit, the Biosource standard were reconstituted and diluted in Assay Diluent. The WHO standards were spiked into 1 ml of NHS and diluted further with Assay Buffer. The assay was performed according to the Upstate protocol as described above.

### Cross-testing of kit standards and comparison to WHO reference standards

To get insight how well the three different kit standards correlate with each other and the WHO standard, the following experimental set-up was applied: as example for the Linco kit, the reconstituted standards from Linco, Upstate and Biosource, respectively, were diluted with Linco Assay Buffer according to their own (Upstate, Linco, and Biosource, respectively) kit manual. The WHO standards were spiked into, and further diluted with, the Linco Assay Buffer. The assay was performed according to the Linco protocol.

In analogous experiments, the above outlined set-up was applied to the Upstate and the Biosource kit and the individual assay was subsequently performed according to the Upstate protocol and Biosource protocol.

### Serum samples from rhesus monkeys

Safety, tolerability and immunogenicity of multiple subcutaneous injections of Vela402 were evaluated in vaccination studies in Rhesus monkeys. Vela402 (previously IGN402) is a multi-component synthetic cancer vaccine containing SialylTn carbohydrate tumor-associated antigen epitopes coupled to a highly immunogenic protein carrier and formulated in a highly immunogenic formulation with QS-21 adjuvant (Kircheis et al. 2006; Kircheis et al. 2007). The animal study was performed under controlled and documented conditions in accordance with animal health care standards at Biotest Ltd., Konarovice, Czech Republic. Healthy adult Rhesus monkeys, four animals per group, were vaccinated with four initial immunizations on days 1, 15, 29, and 57. Pre-sera and immune sera taken at day 43 were analyzed for cytokines (IL-2, IL-4, TNFα, IFNγ) by xMAP Multiplex technology (Luminex) using the three kits (Biosource, Linco and Upstate) according to the manufacturer’s protocol.

## Results

**Figure 1:**
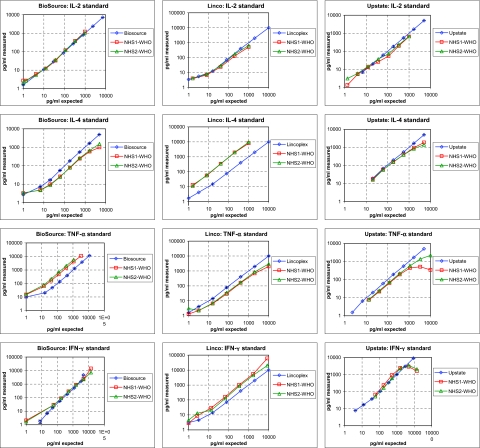
Standard comparison. Standards provided within each kit were diluted according to the protocol with matrix diluent. The WHO standards were spiked into two different normal human sera from healthy donors (designated “NHS1-WHO” and “NHS2-WHO”) and diluted with matrix diluent. Panels grouped ‘in line’ show the indicated cytokine determined with three different kits; panels grouped ‘in row’ show all cytokines determined with one kit.

To determine the accuracy of the standards of each kit, we measured the standards provided with the respective kit, e.g. Linco standards (diluted in serum matrix diluent) in the Linco kit, and compared them to WHO standards spiked into similarly diluted normal human serum (NHS) matrix from two healthy donors. As can be seen in Figure 1 the read-out obtained with the IL-2 standards provided by all kits were in accordance with the WHO standard. In contrast, the IL-4 standard from Linco was greatly (more than 1 log) underestimated in the Linco kit compared to the WHO standards (Fig. 1) which would result e.g. in a dramatic overestimation of the IL-4 titers in serum. For TNFα, the Biosource standard was found to be underestimated in the Biosource kit (Fig. 1). The Linco TNFα standards performed slightly better than the WHO standard resulting in a slight underestimation of the TNFα serum content suggesting that the values measured are, in fact, higher. With the Upstate kit, the WHO calibration curve generated showed at its highest concentration a so-called “hook-effect” which is often observed in biological assays with samples at high serum concentration and which may be due to inhibitory factors found at high serum concentrations. Another explanation for the hook effect might be an ‘over-coating’ of the Upstate beads with the capture antibody. Finally, IFNγ measurements revealed a good alignment with the WHO and the Biosource standards, a slight underestimation of the Linco standards and good alignment with the WHO standard, and a “hook-effect” at high concentrations, in the Upstate kit. The possibility that the isotype of the capture/detection antibody pairs is responsible for the reported differences can be ruled out because for at least 3 cytokines (IL-2, TNFα and IFNγ) these isotypes are identical at least in two kits (Linco and Biosource kit—data for the Upstate kit were not available).

**Figure 2:**
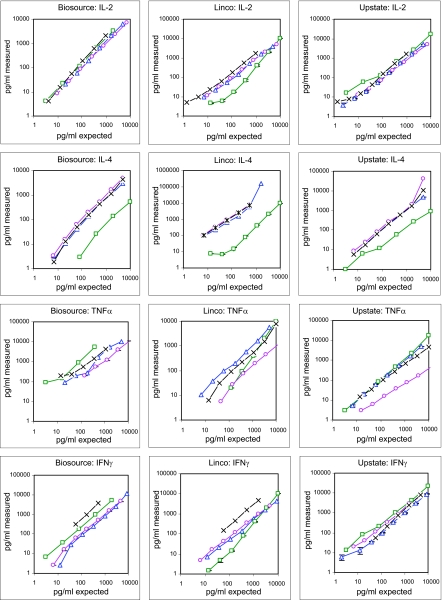
Cross-testing of kit standards and comparison to WHO reference standards. Standards provided within the Upstate (blue triangles), Linco (green squares), Biosource (magenta circles) kit and WHO reference standards (black crosses) were diluted in assay buffer of one kit and measured according to the kit’s protocol.

Next we determined how accurate each kit measures its own standards and the standards provided by the other kits as compared to the WHO standard (Fig. 2). Regarding IL-2, all standards were recovered equally well with the Biosource and the Upstate kit. In contrast, within the Linco kit, all standards were overestimated when compared to Linco IL-2 standard. For IL-4, the Linco standard was greatly underestimated compared to the Biosource and the Upstate standards in all three assays. The TNFα standard from Linco was somewhat overestimated in the Biosource kit but reasonably quantified with the other kits. In contrast, the Biosource standard was underestimated when measured in the Linco and the Upstate kit, respectively. Noteworthy, for IFNγ the WHO calibrator performed better than all other standards when measured with the Biosource and the Linco kit. With the Upstate kit, no differences between kit standards and WHO standards were found. In summary, the cross-testing of the three kit standards revealed that the Biosource standards worked excellently for IL-2 and IL-4 in all three kits but the TNFα and IFNγ the standards from Biosource were underestimated in the Linco and the Upstate kits. Of all standards tested, the Linco standards showed the largest deviations—even when tested in their own kit, only TNFα correlated with the WHO reference. In contrast, the Upstate standards performed equally well in all three kits and the obtained read-out always correlated with the WHO standard indicating that this kit is best suited for reliable cytokine determination.

**Figure 3:**
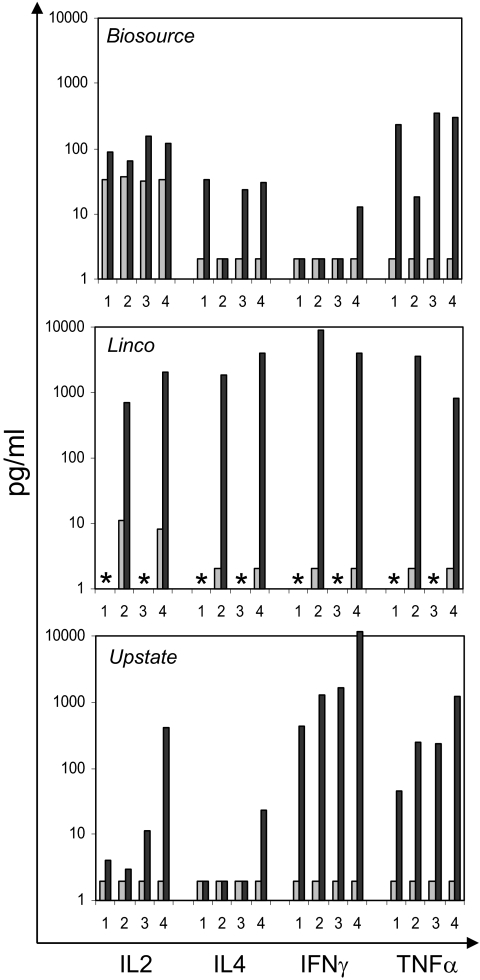
Kit comparison: Serum samples from Rhesus monkeys before and after immunization with Vela402 conjugate vaccine were analyzed for IL-2, IL-4, IFNγ and TNFα using kits from three manufactures. Pre-serum levels are shown as light grey bars, immune serum levels are shown in dark grey. Asterisks: sample not measured. The Mean values of duplicate measurements are shown.

We finally applied the three kits to “real serum sample” measurements, i.e. serum obtained from a vaccination study in Rhesus monkeys. The detection of systemic cytokine release after vaccination applying xMAP technology has been recently demonstrated by Kircheis et al. (2007). As shown in Figure 3, depending on the kit used, different amounts of released cytokines were detected after vaccination when compared to the corresponding pre-serum. For IL-2, the Linco kit identified individuals (ID) 2 and 4 with up to 100-fold increased cytokine levels after immunization. In contrast, with the Biosource these two samples were only weakly positive and with the Upstate kit only in ID 4 a response was detected (which was of the same magnitude as measured with the Linco kit). The differences between the various kits were particularly evident when comparing the IL-4 data: while for e.g. ID 4 all three kits detected a significant increase after immunization, for ID 2 only background values were measured with the Biosource and the Upstate kit whereas the Linco assay detected significant amounts in ID 2 AND ID 4 following immunization. Elevated TNFα levels in the immune sera were identified with the all three kits applied. For IFNγ, the Linco kit gave the highest read-out which was partly in agreement with the data generated using the Upstate kit which also identified ID 2 AND ID 4 immune sera as positive—albeit at ~ 10-fold lower levels. In contrast, the Biosource kit did not detect IFNγ in ID 2 but (at marginal levels) in ID 4.

The obtained differences between the kits can not be explained by a difference in the assay sensitivity because the sensitivity of all three kits (as stated by the manufactures) lies roughly in same range (Table 1). A potential reason for the observed differences might be due to a different cross-reactivity of the different antibody pairs with the monkey serum and the human serum, respectively. Based on the extremely high homology of human and monkey INFγ, TNFα, IL-2 and IL-4 such cross-reactivity is expected to be neglectable.

## Discussion

Prior to measurement of the induced cytokine levels of serum samples by xMAP multiplex technology, the performance of kits from three different manufactors (Linco, Biosource and Upstate, respectively) was evaluated. The three kits were tested according to the protocol provided with each kit. Our results revealed striking differences questioning the usefulness of these kits regarding the accurate and reliable determination of the cytokine amounts. In detail, we found a substantial discordance of the supplied standards with the respective WHO standards. In particular, the IL-4 standard provided by the Linco kit was significantly underestimated in all three kits (including the Linco kit) which would result in an overestimation of the IL-4 titers of measured samples when calibrated using the IL-4 standard curve. Furthermore, the Linco IL-2 standard was underestimated in the Linco kit and Biosource TNFα standard was underestimated in the Biosource kit.

In this context, Prabhakar and colleagues found that—when measuring human serum samples with a Linco kit—only the recovery for TNFα was within the expected range but recoveries of IL-2, IL-4, IL-5, IL-10 and IFNγ remained low and showed high variability. They also reached the conclusion that ‘real world’ assay performance has to be evaluated thoroughly when trying to compare the results of different methods. Furthermore, Lash et al. (2006) reported that even the results obtained with two multiplex sandwich ELISA’s and a bead based multiplex assay can not be compared.

Hildesheim et al. have reported that using the Linco kit for IL-6 and IL-8 determination resulted in invariably high read-outs, and for IL-4 amounts extremely low values were measured—these findings led to the exclusion of these cytokines from evaluation. Khan and coworkers have compared two kits from different manufacturers, i.e. Biosource and Linco, for IFNγ and TNFα detection in sera of healthy subjects after endotoxin injection. This study also reported higher levels of TNFα using the Biosource kit compared to the Linco kit and highlighted false positive results. Because determination of cytokines using conventional ELISA kits from commercial sources revealed no significant difference for TNFα levels (Aziz et al. 1999) the possibility exits that xMAP based multiplex technology has not been fully standardized yet.

Our data extend these investigations to a preclinical vaccination study where the induction of cytokines (like IL-2, IL-4, IFNγ, and TNFα)—if measured accurately—might serve as surrogate markers for the assessment of therapeutic efficacy (Panelli et al. 2004; Schiller et al. 1990; Blankenstein, 2005; Kircheis et al. 1992; Schmidt et al. 1995; Yang et al. 2004). Serum samples obtained from Rhesus monkeys before and after immunization with the highly immunogenic vaccine formulation Vela402 were used for cytokine determination. The data from the crossover experiments with standards from different kits compared to WHO standard together with the data obtained from the analysis of serum samples indicated that among the different kits tested, the kit from Upstate appeared to give the most reliable data, since it showed the most overlapping results with the other two kits tested. In contrast, the Linco kit—although possibly providing highest sensitivity, tends to provide overestimated values due to the underestimation of its own standard compared to the WHO and the standards of the other kits. Biosource showed generally the lowest values for cytokine release among the three kits. Therefore, in all our following preclinical studies concerning the Vela402 project cytokine release was measured using the Upstate kit.

Our results suggest that the performance of (at least three) available multiplex kits differs and that therefore initial kit testing prior to a series of measurements might improve the quality of the data. For the measurement of samples from clinical studies we highly recommend the validation of the particular assay (which has to be specified in the clinical study protocol) according to International Conference of Harmonization (ICH) guidelines. Such validation guarantees that the obtained data are valid and can be reported to authorities (especially when the standard curve was recorded using an international—and therefore accepted—standard). Differences due to different kit or reagent lots which are one of the main sources for unexpected results can be identified applying a validated assay.

This validation approach has been performed for the simultaneous measurement of five cytokines using 2000 serum samples from patients with sepsis (Ray et al. 2005). The result showed that for TNFα the number of freeze/thaw cycles must not exceed two—otherwise the recovery is decreasing. The serum samples that were used in our study were frozen/thawed only once. Thus the appropriate storage conditions should guarantee analyte stability and therefore altered analyte(s) are not the explanation for the observed discrepancies between the kits used. Additional critical parameters, like appropriate negative control matrices, have to be identified by a risk assessment procedure that should be based on the known pitfalls of multiplexing described in literature (Whiteside et al. 2002) and the specific patient population studied. Initial experiments have to focus on assessing the influence of serum components (e.g. immunoglobulins, lipids or rheumatoid factors) and the sample dilution on the recovery rate of the various cytokines. It should be noted that, whereas information regarding the antibody isotypes used within the various kits was partly available, no information was given by the manufacturers regarding the specific clones used. This information would be helpful because the clone specific antibodies might show differing avidity/affinity for the antigens, a fact which could influence the quantitation.

In summary, we wish to point out that performing an assay “according to the manufacturer’s instructions” does not necessarily guarantee reliable quantitative data—and even the collection of qualitative data may be questionable. Especially, in the light of the increasing interest in potential serum surrogate parameters indicative for clinical efficacy (Saheb et al. 2007), the results of the present study indicate that cytokine level measurements in human serum have to be interpreted with great caution, especially with respect to the absolute cytokine amounts reported.

## Figures and Tables

**Table 1:** **Assay sensitivity (pg/ml).**Sensitivity is defined according to the Linco datasheet as the “minimum detectable concentration” and within the Upstate datasheet as “two standard deviations above the mean MFIof 20 replicates of the zero standard”. No definition is given within the Biosource datasheet.

	Upstate	Biosource	Linco
IL-2	1	<6	0.9
IL-4	1	<5	4
TNFα	1	<10	3.3
IFNγ	16	<5	1.3
